# A promoter polymorphism rs2075824 within IMPA2 gene affecting the transcription activity: possible relationship with schizophrenia

**DOI:** 10.1111/jcmm.13009

**Published:** 2016-10-17

**Authors:** Jia Li, Sheng Huang, Hui‐Rong Dai, Juan Wang, Li‐Hui Lin, Hui Xiao, Xia Peng, Fei Li, Yu‐Ping Wang, Jian‐Min Yuan, Li Li

**Affiliations:** ^1^ Department of Laboratory MedicineSchool of MedicineShanghai General HospitalShanghai JiaoTong UniversityShanghaiChina; ^2^ Department of Central LaboratoryWuxi Mental Health CenterWuxiJiangsuChina

**Keywords:** myo‐inositol monophosphatase 2, schizophrenia, Han Chinese, SNPs

## Abstract

Previous studies with biological and genetic evidence indicate that the myo‐inositol monophosphatase 2 (*IMPA2*) gene may influence schizophrenia. We performed a genetic association study in Han Chinese cohorts. Five single nucleotide polymorphisms within IMPA2 promoter region (rs971363, rs971362, rs2075824, rs111410794 and rs111610121), as well as one (rs45442994, in intron 1) that was positively associated in another study, were selected for genotyping in 1397 patients with schizophrenia and 1285 mentally healthy controls. Genotype and allele frequencies were assessed by gender stratification. Interestingly, rs2075824 showed a strong association with schizophrenia (*P* = 4.1 × 10^−4^), and the T allele was more frequent in cases than controls [*P* = 5.6 × 10^−5^, OR (95% CI) = 1.26 (1.13–1.41)]. In vitro promoter assay showed that the transcription activity of the T allele promoter was higher than that of the C allele promoter and the T allele of rs2075824 contributed to risk for schizophrenia. By stratifying males and females, we found a gender‐specific association for *IMPA2* and schizophrenia: the T allele of rs2075824 was more frequent in male cases compared with male controls [*P* = 1.4 × 10^−4^, OR (95% CI) = 1.33 (1.15–1.55)]. Our data suggest that a promoter polymorphism of *IMPA2* possibly contributed to risk for schizophrenia by elevating transcription activity in Han Chinese individuals.

## Introduction

Schizophrenia (SCZ) is characterized by a constellation of chronic symptoms including hallucinations, delusions, social and occupational deterioration, and affects approximately 1% of the population worldwide 1, 2. Linkage studies have revealed that SCZ and bipolar disorder share a number of overlapping features and genetic risk variants 3, 4. Bipolar disorder is associated with neurocognitive deficits that persist into euthymia after episode resolution 5. Several meta‐analyses have revealed impairments in the broad domains of attention, processing speed, verbal memory and executive functions, with relative preservation of verbal abilities and intelligence. This pattern of deficits is similar to that in SCZ, although less severe in magnitude 6. One locus, 18p11.2, was identified decades ago as a susceptibility locus for both disorders 7, 8, and has since been confirmed in other studies 9, 10, 11, 12. Myo‐inositol monophosphatase 2 (*IMPA2*) is a candidate gene in this region which was identified by Yoshikawa *et al*. 13. Subsequently, other studies in different ethnic groups verified the relationship between *IMPA2* and SCZ and bipolar disorder 14, 15, 16, 17. Furthermore, Yoon *et al*. reported that *IMPA2* showed gender‐dependent expression differences in the brains of patients with bipolar disorder 18. Recently, Ramsey *et al*. proposed to investigate the gender‐specific etiologies of SCZ 19; however, to our knowledge no study to date has performed a gender‐specific analysis between *IMPA2* and SCZ. This prompted us to examine the contribution of IMPA2 to SCZ in Han Chinese cohorts.

## Materials and methods

### Subjects

1397 unrelated individuals with SCZ and 1285 controls were included in this study. No significant differences were found with respect to population characteristics between the two groups (Table 1). The cases were inpatients or outpatients from Wuxi Mental Health Center identified on the basis of previously established criteria: (*i*) patients met the Diagnostic and Statistical Manual of Mental Disorders, Fourth Edition (DSM‐IV) criteria for SCZ 20, 21, and the diagnoses were confirmed by two independent psychiatrists who reviewed all medical records of the patients, interviewed collateral informants (most often clinical staffs), and used information from recent interviews with the patients; (*ii*) patients had no physical disease or other psychiatric disorder apart from SCZ. Controls were randomly selected from the Shanghai general population and were all interviewed by professional psychiatrists using the Structured Clinical Interview for DSM‐ IV (a diagnostic exam used to determine DSM‐IV disorders, the instrument was designed to be administered by a mental health professional, for example a psychologist or psychiatrist.) to exclude a history of psychiatric disorders, chronic neurological disease, and family history of psychiatric disorders.

**Table 1:** Characteristics of schizophrenia patients and controls

Variable	*n*	Age (mean ± S.D., years)	Gender, female:male
SCZ group	1397	46.7 ± 12.2	556:841
CTR group	1285	44.7 ± 11.1	559:726
*P*‐value	–	0.471	0.052

SCZ: schizophrenia; CTR: control.

The study protocol was approved by the Ethics Committee of Shanghai First People's Hospital, and was conducted in agreement with the Declaration of Helsinki Principles 22. Informed written consent was obtained from all participants. We used the following criteria to evaluate whether the participants had the capacity to consent: (*i*) patient's ability to understand; (*ii*) patient's ability to reason; and (*iii*) patient's ability to make rational decisions. If participants failed to fill out the consent form more than twice, their guardians were asked to fill out the consent form on the patients' behalf.

### DNA extraction

Blood samples were collected from all participants using K_2_EDTA tubes. Genomic DNA was extracted from peripheral leucocytes by standard procedures using a Genomic DNA kit (Axygen, Hangzhou, China) and then stored at −80°C for genotype analysis.

### SNP selection and genotyping

Five single nucleotide polymorphisms (SNPs) within IMPA2 promoter region (rs971363, rs971362, rs2075824, rs111410794 and rs111610121), as well as rs45442994 in intron 1 were selected for case–control analysis. Genotyping of six polymorphisms was performed using PCR and ligase detection reaction (LDR). PCR primers and LDR probes were synthesized by Invitrogen (Shanghai, China) and the sequences are available (see Tables S1 and S2). For PCR, 1 μl of each DNA sample was added to 19 μl of PCR mixture containing 1× PCR buffer, 1× Q‐Solution (Qiagen, Hilden, Germany), 3 mmol/l MgCl_2_, 0.2 mmol/l of each dNTP, 50 pmol/μl primers, and 1 U of HotStar Taq polymerase (Qiagen). PCR cycling conditions were: pre‐denaturation at 95°C for 2 min., denaturation at 94°C for 30 sec., anneal at 56°C for 90 sec., and extend at 72°C for 60 sec., the application was performed for 35 cycles, and a final 10 min. extension step at 65°C. PCR products were observed by agarose gel electrophoresis (3.0%), visualized with ImageMaster VDS (Pharmacia Biotech, Piscataway, NJ, USA), and then used as templates in LDR. LDR reactions were carried out in 10 μl reaction mixtures consisting of 1× Buffer, 2 pmol/μl Probe mix, 2 U NEB Taq DNA ligase, and 100 ng/μl PCR product. The reaction program was performed as follows: an initial heating at 95°C for 2 min., followed by 40 cycles (94°C for 15 sec. and 50°C for 25 sec.). The products were submitted for sequencing on a PRISM 3730 (ABI) sequencer, and the results were analysed with GeneMapper software, version 3.3 (Applied Biosystems Incorporation, Foster City, CA, USA).

### Plasmid construction

Reporter plasmids carrying luciferase gene under the control of IMPA2 promoter with rs2075824 C (pGL3‐Basic‐IMPA2) and rs2075824 T (pGL3‐Basic‐IMPA2‐Mut) were constructed. Rs2075824 T allele promoter and C allele promoter were synthesized by Shanghai Novo Biotechnology Co., Ltd (Xuhui District, Shanghai, China) according to the sequences provided in another report by Ohnishi *et al*. 16 and were verified by sequencing.

The synthesized IMPA2 promoters were directly cloned into MluI/XhoI site of pGL3‐Basic of vector. The structure of each construct was verified by sequencing. The vector pGL3‐control containing the SV40 promoter was used as a positive control.

### Cell culture, transfection and luciferase assay

SH‐SY‐5Y (neuroblastoma cell line) was cultured in DMEM (Gibco, Gaithersburg, MD, USA) containing 10% heat‐inactivated foetal bovine serum (Gibco, Gaithersburg, MD, USA) in 10 cm culture dishes and were passaged at 60–70% confluence (1.8 × 10^5^ cells/well) onto a 24‐well plate, 1 day before transfection. Transfections were performed using Lipofectamine 2000 (Invitrogen) following the manufacturer's instructions. 1.6 μg of plasmid DNA and 4 μl of Lipofectamine 2000 were mixed in 200 μl of OPTI‐DMEM. After 20‐min incubation, 1 ml of OPTI‐DMEM was added to individual wells and the Lipofectamine 2000/plasmid mixture was then added to each well containing cells. The plate was placed in a CO_2_ incubator at 37°C. Medium was replaced 4–6 hrs after transfection. We performed the transcriptional assay using Renilla Luciferase Assay System (Promega, Madison, WI, USA). Transfected cells were washed with PBS 48 hrs after transfection and incubated in 200 μl of cell lysis buffer for 1 hr on ice. Next, 20 μl firefly was added into 20 μl cell lysate. The dual luciferase assay was carried out using a Modulus luminometer (Turner Biosystems, Sunnyvale, CA, USA).

### Statistical analysis

Testing of genotypes for Hardy–Weinberg equilibrium (HWE) in all participants was determined by exact test included in the PLINK software package, version 1.07 23. The threshold for significant deviation from HWE was set as 0.01. The differences in allele and genotype frequencies were analysed by use of the chi‐square text in PLINK software. The test of significance was two‐tailed, and alpha was set at 0.05. Bonferroni correction was applied for multiple comparisons of genotype frequencies, and alpha was set at 0.017. A logistic regression adjusted for age and gender was applied to evaluate how these factors influence the distribution of *IMPA2* polymorphisms. Effect of rs2075824 on the transcription activity of IMPA2 promoter was conducted with a paired *t*‐test. All tests were two‐tailed and statistical significance was defined as *P* < 0.05.

## Results

### IMPA2 SNPs and schizophrenia associations

All the six analysed SNPs were in HWE in the SCZ and control groups (*P* = 0.38 for rs971363, 0.27 for rs971362, 0.09 for rs2075824, 0.28 for rs111410794, 0.31 for rs111610121, 0.17 for rs45442994). As presented in Table 2, the genotype frequencies of rs2075824 were significantly different between SCZ patients and control groups (*P* = 4.1 × 10^−4^); the frequency of the T allele was higher in SCZ patients than in the control group (*P* = 5.6 × 10^−5^); after a 10,000 permutation correction, the differences were still significant (*P* = 1.4 × 10^−4^ for genotype frequencies & *P* = 1.1 × 10^−4^ for allele frequencies). By performing Bonferroni correction (α = 0.017), the genotype frequencies of rs2075824 were significantly differ between SCZ patients and control groups. Furthermore, the polymorphism increased the risk of development of SCZ (OR = 1.26, 95% CI = 1.13–1.41). The rare allele T of rs2075824 contributed to disease susceptibility. There were no other significant disease risk associations.

**Table 2:** Association analysis of the six selected SNPs in 1397 patients with schizophrenia and 1285 mentally healthy controls

Test model:SNP(A1/A2)*	SCZ freq. (%)	CTR freq. (%)	*P*‐value†	*P*‐value‡	OR (95% CI)
rs971363(G;T)					
Genotype:GG/GT/TT	9.2/40.6/50.3	8.7/41.9/49.4	0.78	0.86	1
Allele:G/T	29.5/70.5	29.6/70.4	0.88	0.86	0.99 (0.88–1.11)
rs971362(T;G)					
Genotype:TT/GT/GG	10.7/46.0/43.4	11.7/46.2/42.1	0.65	0.53	1
Allele:T/G	33.6/66.4	34.8/65.2	0.38	0.35	0.95 (0.85–1.06)
rs2075824(T;C)					
Genotype:TT/CT/CC	15.4/44.9/39.7	11.4/42.4/46.2	**4.1 × 10** ^**−4**^	**1.4 × 10** ^**−4**^	1
Allele:T/C	37.8/62.2	32.6/67.4	**5.6 × 10** ^**−5**^	**1.1 × 10** ^**−4**^	1.26 (1.13–1.41)
rs111410794(C;T)					
Genotype:CC/CT/TT	16.6/45.5/37.9	14.3/46.2/39.5	0.25	0.75	1
Allele:C/T	39.4/60.6	37.4/62.6	0.15	0.53	0.92 (0.83–1.03)
rs111610121(A;G)					
Genotype:AA/AG/GG	8.7/38.5/52.8	7.9/37.0/55.1	0.45	0.33	1
Allele:A/G	28.0/72.0	26.4/73.6	0.21	0.21	0.92 (0.82–1.04)
rs45442994(A;G)					
Genotype:AA/AG/GG	4.8/37.1/58.1	5.8/33.5/60.6	0.11	0.60	1
Allele:A/G	23.3/76.7	22.6/77.4	0.53	0.53	1.04 (0.92–1.18)

*A1/A2, indicates minor allele/major allele. ^†^Significant *P*‐values (<0.05) are in boldface. ^‡^Global *P*‐value after 10,000 permutation correction for multiple testing. SCZ: schizophrenia; CTR: control; OR, odd ratio; 95% CI, 95% confidence interval.

### Gender‐specific association

To examine whether gender‐specific differences are involved in the association, we analysed our data by stratifying males and females. We found that rs2075824 had significant allelic (*P* = 1.4 × 10^−4^) and genotypic (*P* = 9.5 × 10^−4^) associations with SCZ in all males (Table 3) but not in all females (Table 4). To verify these positive results, a 10,000 permutations test (Tables 2, 3, 4) was conducted and supports these associations. Logistic regression models adjusted for age and gender (*P* = 8.28 × 10^−5^, OR = 1.25, 95% CI = 1.12–1.40) confirmed that the ‘T’ allele of rs2075824 is consistently associated with an increased risk of SCZ (Table 5).

**Table 3:** Association analysis of the six selected SNPs in males with schizophrenia and healthy male controls

Test model:SNP(A1/A2)*	SCZ freq. (%)	CTR freq. (%)	*P*‐value†	*P*‐value‡	OR (95% CI)
rs971363(G;T)					
Genotype:GG/GT/TT	8.0/41.7/50.3	9.4/43.1/47.5	0.43	0.60	1
Allele:G/T	28.8/71.2	30.9/69.1	0.20	0.13	0.91 (0.78–1.06)
rs971362(T;G)					
Genotype:TT/GT/GG	10.3/47.9/41.7	11.6/49.6/38.8	0.46	0.62	1
Allele:T/G	34.3/65.7	36.4/63.6	0.23	0.75	0.91 (0.79–1.06)
rs2075824(T;C)					
Genotype:TT/CT/CC	16.1/45.2/38.8	11.2/41.9/47.0	**9.5 × 10** ^**−4**^	**1.0 × 10** ^**−3**^	1
Allele:T/C	38.6/61.4	32.1/67.9	**1.4 × 10** ^**−4**^	**1.5 × 10** ^**−4**^	1.33 (1.15–1.55)
rs111410794(C;T)					
Genotype:CC/CT/TT	16.6/45.6/37.8	14.3/46.2/39.5	0.43	0.35	1
Allele:C/T	39.4/60.6	37.4/62.6	0.25	0.26	0.92 (0.80–1.06)
rs111610121(A;G)					
Genotype:AA/AG/GG	8.8/38.5/52.7	8.0/36.9/55.1	0.61	0.67	1
Allele:A/G	28.1/71.9	26.4/73.6	0.32	0.38	0.92 (0.79–1.08)
rs45442994(A;G)					
Genotype:AA/AG/GG	4.4/36.9/58.7	5.6/31.4/62.9	0.06	0.09	1
Allele:A/G	22.8/77.2	21.3/78.7	0.32	0.38	1.09 (0.92–1.29)

*A1/A2, indicates minor allele/major allele. ^†^Significant *P*‐values (<0.05) are in boldface. ^‡^Global *P*‐value after 10,000 permutation correction for multiple testing. SCZ: schizophrenia; CTR: control; OR, odd ratio; 95% CI, 95% confidence interval.

**Table 4:** Association analysis of the six selected SNPs in females with schizophrenia and healthy female controls

Test model:SNP(A1/A2)*	SCZ freq. (%)	CTR freq. (%)	*P*‐value	*P*‐value†	OR (95% CI)
rs971363(G;T)					
Genotype:GG/GT/TT	11.0/38.8/50.2	7.9/40.3/51.9	0.21	0.22	1
Allele:G/T	30.4/69.6	28.0/72.0	0.21	0.21	1.13 (0.94–1.35)
rs971362(T;G)					
Genotype:TT/GT/GG	11.2/43.0/45.9	11.8/41.9/46.3	0.90	0.85	1
Allele:T/G	32.6/67.4	32.7/67.3	0.96	1.00	1.00 (0.83–1.19)
rs2075824(T;C)					
Genotype:TT/CT/CC	14.4/44.4/41.2	11.6/43.1/45.3	0.25	0.34	1
Allele:T/C	36.6/63.4	33.2/66.8	0.09	0.14	1.16 (0.98–1.38)
rs111410794(C;T)					
Genotype:CC/CT/TT	16.5/45.5/37.9	14.3/46.3/39.4	0.58	0.67	1
Allele:C/T	39.3/60.7	37.5/62.5	0.38	0.40	0.93 (0.78–1.10)
rs111610121(A;G)					
Genotype:AA/AG/GG	8.6/38.5/52.9	7.9/37.0/55.1	0.74	0.86	1
Allele:A/G	27.9/72.1	26.4/73.6	0.44	0.50	1.93 (0.77–1.12)
rs45442994(A;G)					
Genotype:AA/AG/GG	5.4/37.4/57.2	6.1/36.3/57.6	0.85	1.00	1
Allele:A/G	24.1/75.9	24.2/75.8	0.97	0.86	1.00 (0.82–1.22)

*A1/A2, indicates minor allele/major allele. ^†^Global *P*‐value after 10,000 permutation correction for multiple testing. SCZ: schizophrenia; CTR: control; OR, odd ratio; 95% CI, 95% confidence interval.

**Table 5:** Risk estimates using a logistic regression model for six SNPs in *IMPA2*

SNP	Allele	Total (*n* = 2682)		Male (*n* = 1567)		Female (*n* = 1115)	
		*P*‐value*	OR (95% CI)	*P*‐value†	OR (95% CI)	*P*‐value†	OR (95% CI)
rs971363	G	0.88	0.99 (0.88–1.11)	0.17	0.90 (0.77–1.05)	0.19	1.13 (0.94–1.35)
rs971362	T	0.37	0.95 (0.85–1.06)	0.18	0.90 (0.77–1.05)	0.97	1.00 (0.84–1.19)
rs2075824	T	8.28 × 10^−5^	1.25 (1.12–1.40)	1.05 × 10^−4^	1.33 (1.15–1.54)	0.14	1.14 (0.96–1.35)
rs111410794	C	0.15	1.08 (0.06–0.97)	0.25	1.09 (0.94–1.25)	0.38	1.08 (0.91–1.28)
rs111610121	A	0.21	1.08 (0.96–1.21)	0.32	1.08 (0.93–1.26)	0.44	1.08 (0.90–1.29)
rs45442994	A	0.53	1.04 (0.92–1.18)	0.28	1.10 (0.93–1.30)	0.86	0.98 (0.81–1.19)

*P‐values in the logistic regression model, adjusted by gender and age. ^†^ *P*‐values in the logistic regression model, adjusted by age.

The other polymorphisms did not display a significant difference in the distributions of genotypes and alleles between the case and control group, even when stratified by gender (Tables 2, 3, 4). Linkage disequilibrium estimation for all SNPs was not performed because only rs2075824 yielded a significant association.

### The rs2075824 T/C polymorphism affecting the transcription activation of IMPA2 promoter

**Figure 1:**
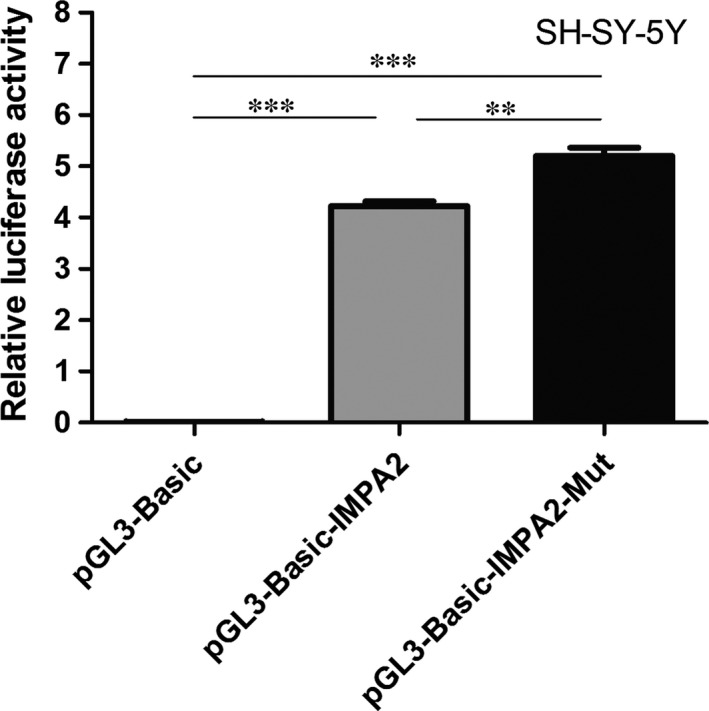
Effect of rs2075824 T/C polymorphism on the transcription activity of IMPA2 promoter. SH‐SY‐5Y cells were transiently transfected with pGL3‐Basic‐IMPA2 (IMPA2 promoter with rs2075824 C), pGL3‐Basic‐IMPA2‐Mut (IMPA2 promoter with rs2075824 T), or pGL3‐Basic. The relative luciferase activity is represented as the ratio of the activity to that of pGL3‐Basic. Data represent the average ± S.D. of triplicate samples. A representative result of three independent experiments is shown. **P < 0.01; ***P < 0.005, as determined by a paired *t*‐test.

To evaluate the effects of rs2075824 T/C in IMPA2 promoter region on transcription activity, we prepared two reporter plasmids carrying the luciferase gene under the control of the IMPA2 promoter. Each of the reporter plasmids was transfected into the human neuroblastoma cell line SH‐SY‐5Y. As shown in Figure 1, luciferase activity derived from the rs2075824 T promoter was higher than that from the rs2075824 C promoter (*P* < 0.01), suggesting that the rs2075824 T promoter may result in enhanced promoter activity in the brain.

## Discussion

Myo‐inositol monophosphatase 2 has been considered a functional candidate gene for SCZ because it was presumed to be involved in the protein kinase C signalling pathway, which regulates multiple neuronal processes implicated in mood regulation 24. It encodes myo‐inositol monophosphatase 2 (IMPase), which catalyses the final step in the inositol recycling by dephosphorylating myo‐inositol monophosphate to regenerate free inositol 17, 25. An abnormality of inositol availability has been observed in specific brain regions of patients with SCZ 26. In addition, IMPase is a putative target for lithium 27, which has been used in psychiatric clinical treatment for over a century.

Several previous studies have investigated the genetic association and function of *IMPA2* 14, 15, 16, 18. Sjoholt *et al*. described a family‐based association study in a Palestinian‐Arab population in which SNPs in the promoter region of *IMPA2* were associated with bipolar disorder 15. The association was replicated by Ohnishi *et al*. in a Japanese population, and was further substantiated by functional studies that suggested transcriptional effects of a promoter haplotype *in vitro* 16. The only association study between *IMPA2* and SCZ was conducted by Yoshikawa *et al*. in Japanese individuals; the SNPs rs111610121 (in the promoter region), rs2075825 (in exon 6), and rs45442994 (in intron 1) were positively associated with the disorder 14.

To assess the role of *IMPA2* in SCZ in Han Chinese, we carried out a genetic association study. In this study, a total of six SNPs were genotyped for association analyses in 1397 patients with SCZ and 1285 controls. We observed that rs2075824 was significantly associated with SCZ (*P* = 1.4 × 10^−4^); even after correcting for multiple testing and adjusting for gender and age, the genetic effect on SCZ remained significant. In addition, prior studies revealed the same SNP contributed to risk for bipolar disorder in Japanese and Palestinian Arab individuals. Indeed, *IMPA2* may participate in the development of SCZ and bipolar disorder and its promoter may be a hot spot harbouring *IMPA2* variants. However, whether/how the variation affected IMPase expression and inositol synthesis and lead to the SCZ phenotype remains to be explored. This prompted us to examine whether rs2075824 of IMPA2 contributed to risk for SCZ by elevating transcription activity in Han Chinese. Our results suggest that the T allele of rs2075824 was a risk factor for SCZ and may result in enhanced promoter activity in the brain; however, allele‐specific expression studies are needed to determine the results and illuminate the biological mechanisms. According to the functional researches on other loci or diseases reported before 16, 18, the mechanisms maybe the differences in binding affinity of alleles with transcription factors or by interacting with other genes nearby.

According to NCBI database, rs2075824 is very common in CHB (Han Chinese in Beijing, China) + JPI (Japanese in Tokyo, Japan) and CEU (Utah residents with ancestry from northern and western Europe), the minor allele frequencies are 0.5 and 0.16, respectively (http://www.ncbi.nlm.nih.gov/projects/SNP/snp_ref.cgi?rs=2075824). However, to our knowledge, around 30 SCZ‐associated loci have been identified through GWAS; no association of rs2075824 with SCZ was observed in other populations. The most important limiting factor is the sample size.

The relatively small sample size is also a limitation of our study; another limitation is the incomplete phenotypic characterization of the population. From the beginning of participant collection, we tried to collect more participants and more characteristics (age, gender, familial liability to psychotic, illness characteristics, index episode symptoms, response to treatment, socioeconomic or other characteristics, *etc*.), however, considering the characteristics of SCZ patients (①Most of the participants are outpatients who have great mobility and are difficult to track; ②The treatment compliances of SCZ patients were so poor that medications are not standardized), it is difficult to achieve. However, the results of this article sparked us to further study. Currently, we are trying to investigate the biological mechanism of the rise in transcriptional activity *in vitro*, so as to make up the shortcomings above.

In the present study, we also detected a gender‐specific association for IMPA2 and SCZ contributing to the hypothesis that there are differences between men and women regarding the etiology of SCZ 3. However, this result needs to be replicated in different independent samples, since there is a possibility of population bias, the limited sample size for individual genders is not large enough, and a gender‐genotype interaction has not been formally tested 28. Yoshikawa *et al*. discovered an association between rs111610121 and rs45442994 with SCZ in Japanese individuals; this association was not replicated in our Han Chinese population. The discordance may stem from the ethnic differences exist between Chinese and Japanese peoples or the relatively small sample size in this or previous studies. Hence, there still needs further large‐scale replication studies and functional investigation.

## Conclusions

In summary, this study found that the T allele of rs2075824 in *IMPA2* promoter region possibly contributed to risk for SCZ in Han Chinese individuals by elevating transcription, providing evidence for the hypothesis that some susceptibility may be common to both SCZ and bipolar disorder. Furthermore, the findings imply a putative, gender‐dependent relationship between the *IMPA2* gene and SCZ. Additional functional analyses are necessary to fully elucidate the effects of *IMPA2* polymorphisms and their implication on inositol pathway regulation.

## Conflicts of interest

The authors declare that they have no conflicts of interest.

## References

[1] Mueser KT , McGurk SR . Schizophrenia. Lancet. 2004; 363: 2063–72. 15207959 10.1016/S0140-6736(04)16458-1

[2] Yuan J , Jin C , Qin HD , *et al* Replication study confirms link between TSPAN18 mutation and schizophrenia in Han Chinese. PLoS ONE. 2013; 8: e58785. 23505562 10.1371/journal.pone.0058785 PMC3591373

[3] Power RA , Kyaga S , Uher R , *et al* Fecundity of patients with schizophrenia, autism, bipolar disorder, depression, anorexia nervosa, or substance abuse *vs* their unaffected siblings. JAMA Psychiatry. 2013; 70: 22–30. 23147713 10.1001/jamapsychiatry.2013.268

[4] Lichtenstein P , Yip BH , Bjork C , *et al* Common genetic determinants of schizophrenia and bipolar disorder in Swedish families: a population‐based study. Lancet. 2009; 373: 234–9. 19150704 10.1016/S0140-6736(09)60072-6 PMC3879718

[5] Conus P , Macneil C , McGorry PD . Public health significance of bipolar disorder: implications for early intervention and prevention. Bipolar Disord. 2014; 16: 548–56. 24127825 10.1111/bdi.12137

[6] Bourne C , Aydemir O , Balanza‐Martinez V , *et al* Neuropsychological testing of cognitive impairment in euthymic bipolar disorder: an individual patient data meta‐analysis. Acta Psychiatr Scand. 2013; 128: 149–62. 23617548 10.1111/acps.12133

[7] Berrettini WH , Ferraro TN , Goldin LR , *et al* Chromosome 18 DNA markers and manic‐depressive illness: evidence for a susceptibility gene. Proc Natl Acad Sci USA. 1994; 91: 5918–21. 8016089 10.1073/pnas.91.13.5918 PMC44108

[8] Schwab SG , Hallmayer J , Lerer B , *et al* Support for a chromosome 18p locus conferring susceptibility to functional psychoses in families with schizophrenia, by association and linkage analysis. Am J Hum Genet. 1998; 63: 1139–52. 9758604 10.1086/302046 PMC1377479

[9] Bowen T , Kirov G , Gill M , *et al* Linkage studies of bipolar disorder with chromosome 18 markers. Am J Med Genet. 1999; 88: 503–9. 10490707

[10] Detera‐Wadleigh SD , Badner JA , Berrettini WH , *et al* A high‐density genome scan detects evidence for a bipolar‐disorder susceptibility locus on 13q32 and other potential loci on 1q32 and 18p11.2. Proc Natl Acad Sci USA. 1999; 96: 5604–9. 10318931 10.1073/pnas.96.10.5604 PMC21907

[11] Nothen MM , Cichon S , Rohleder H , *et al* Evaluation of linkage of bipolar affective disorder to chromosome 18 in a sample of 57 German families. Mol Psychiatry. 1999; 4: 76–84. 10089014 10.1038/sj.mp.4000454

[12] Mukherjee O , Meera P , Ghosh S , *et al* Evidence of linkage and association on 18p11.2 for psychosis. Am J Med Genet B Neuropsychiatr Genet. 2006; 141B: 868–73. 16941653 10.1002/ajmg.b.30363

[13] Yoshikawa T , Turner G , Esterling LE , *et al* A novel human myo‐inositol monophosphatase gene, IMP.18p, maps to a susceptibility region for bipolar disorder. Mol Psychiatry. 1997; 2: 393–7. 9322233 10.1038/sj.mp.4000325

[14] Yoshikawa T , Kikuchi M , Saito K , *et al* Evidence for association of the myo‐inositol monophosphatase 2 (IMPA2) gene with schizophrenia in Japanese samples. Mol Psychiatry. 2001; 6: 202–10. 11317223 10.1038/sj.mp.4000835

[15] Sjoholt G , Ebstein RP , Lie RT , *et al* Examination of IMPA1 and IMPA2 genes in manic‐depressive patients: association between IMPA2 promoter polymorphisms and bipolar disorder. Mol Psychiatry. 2004; 9: 621–9. 14699425 10.1038/sj.mp.4001460

[16] Ohnishi T , Yamada K , Ohba H , *et al* A promoter haplotype of the inositol monophosphatase 2 gene (IMPA2) at 18p11.2 confers a possible risk for bipolar disorder by enhancing transcription. Neuropsychopharmacology. 2007; 32: 1727–37. 17251911 10.1038/sj.npp.1301307

[17] Yoshikawa T , Padigaru M , Karkera JD , *et al* Genomic structure and novel variants of myo‐inositol monophosphatase 2 (IMPA2). Mol Psychiatry. 2000; 5: 165–71. 10822344 10.1038/sj.mp.4000688

[18] Yoon IS , Li PP , Siu KP , *et al* Altered IMPA2 gene expression and calcium homeostasis in bipolar disorder. Mol Psychiatry. 2001; 6: 678–83. 11673796 10.1038/sj.mp.4000901

[19] Ramsey JM , Schwarz E , Guest PC , *et al* Distinct molecular phenotypes in male and female schizophrenia patients. PLoS ONE. 2013; 8: e78729. 24244349 10.1371/journal.pone.0078729 PMC3823995

[20] Hasin D , Hatzenbuehler ML , Keyes K , *et al* Substance use disorders: Diagnostic and Statistical Manual of Mental Disorders, fourth edition (DSM‐IV) and International Classification of Diseases, tenth edition (ICD‐10). Addiction. 2006; 101 (Suppl. 1): 59–75. 16930162 10.1111/j.1360-0443.2006.01584.x

[21] Maj M . Critique of the DSM‐IV operational diagnostic criteria for schizophrenia. Br J Psychiatry. 1998; 172: 458–60. 9828982 10.1192/bjp.172.6.458

[22] Carlson RV , Boyd KM , Webb DJ . The revision of the Declaration of Helsinki: past, present and future. Br J Clin Pharmacol. 2004; 57: 695–713. 15151515 10.1111/j.1365-2125.2004.02103.x PMC1884510

[23] Purcell S , Neale B , Todd‐Brown K , *et al* PLINK: a tool set for whole‐genome association and population‐based linkage analyses. Am J Hum Genet. 2007; 81: 559–75. 17701901 10.1086/519795 PMC1950838

[24] Abrial E , Lucas G , Scarna H , *et al* A role for the PKC signaling system in the pathophysiology and treatment of mood disorders: involvement of a functional imbalance? Mol Neurobiol. 2011; 44: 407–19. 21983961 10.1007/s12035-011-8210-4

[25] Atack JR . Inositol monophosphatase, the putative therapeutic target for lithium. Brain Res Brain Res Rev. 1996; 22: 183–90. 8883919

[26] Shimon H , Sobolev Y , Davidson M , *et al* Inositol levels are decreased in postmortem brain of schizophrenic patients. Biol Psychiatry. 1998; 44: 428–32. 9777173 10.1016/s0006-3223(98)00071-7

[27] Berridge MJ , Downes CP , Hanley MR . Neural and developmental actions of lithium: a unifying hypothesis. Cell. 1989; 59: 411–9. 2553271 10.1016/0092-8674(89)90026-3

[28] Patsopoulos NA , Tatsioni A , Ioannidis JP . Claims of sex differences: an empirical assessment in genetic associations. JAMA. 2007; 298: 880–93. 17712072 10.1001/jama.298.8.880

